# A Dietary Computer

**Published:** 1918-01

**Authors:** Ruth Raymond


**A Dietary Computer.** Amy Elizabeth Pope, San Francisco. G. P. Putnam's Sons, New York. 156 pages, $1.25.

In these days of greatly awakened interest in Dietetics, due to the impetus caused by the food problem under war conditions, this book will prove to be very practical for nurses working in conjunction with physicians on special diets, or for the laity who desire to use scientific principles in planning the family dietary. It includes a statement with regard to the general principles underlying food requirements under different conditions, with a simple method of calculating the fuel value of the necessary menus and lists of foods relatively rich in special constituents. There are also tables showing the chemical composition and caloric value of common foods and beverages, which include, in the case of the more commonly used foods, the size of a single portion, and tables showing the cost, chemical composition and caloric value of some special diabetic foods, with a table showing the average relative carbohydrate content of food. The caloric values of the more common species of invalid cookery are given.—Ruth Raymond, B. S.

